# Climate change will disproportionally affect the most genetically diverse lineages of a widespread African tree species

**DOI:** 10.1038/s41598-022-11182-z

**Published:** 2022-04-29

**Authors:** Paul T. Lyam, Joaquín Duque-Lazo, Frank Hauenschild, Jan Schnitzler, Alexandra N. Muellner-Riehl, Michelle Greve, Henry Ndangalasi, Annerine Myburgh, Walter Durka

**Affiliations:** 1 Department of Molecular Evolution and Plant Systematics and Herbarium (LZ), Institute of Biology, Leipzig University, Johannisallee 21-23, 04103 Leipzig, Germany; 2 German Centre for Integrative Biodiversity Research (iDiv) Halle-Jena-Leipzig, Puschstrasse 4, 04103 Leipzig, Germany; 3 National Centre for Genetic Resources and Biotechnology, NCRI complex, Moor Plantation, P.M.B 5282, Ibadan, Nigeria; 4 Department of Community Ecology (BZF), Helmholtz Centre for Environmental Research–UFZ, Theodor-Lieser-Str. 4, 06120 Halle, Germany; 5 Agresta SCoop. C/ Duque de Fernán Núñez, 2, 1°, 28012 Madrid, Spain; 6 Department of Plant and Soil Sciences, University of Pretoria, Pretoria, 0002 South Africa; 7 Department of Botany, University of Dar Es Salaam, P.O. Box 35060, Dar es Salaam, Tanzania

**Keywords:** Biogeography, Genetic variation, Ecological modelling

## Abstract

Global climate change is proceeding at an alarming rate with major ecological and genetic consequences for biodiversity, particularly in drylands. The response of species to climate change may differ between intraspecific genetic groups, with major implications for conservation. We used molecular data from 10 nuclear and two chloroplast genomes to identify phylogeographic groups within 746 individuals from 29 populations of *Senegalia senegal*, a savannah tree species in sub-Saharan Africa. Three phylogroups are identified corresponding to Sudano-Sahelian, Zambezian and Southern African biogeographic regions in West, East and Southern Africa. Genetic diversity was highest in Southern and Zambesian and lowest in the Sudano-Sahelian phylogroups. Using species distribution modeling, we infer highly divergent future distributions of the phylogroups under three climate change scenarios. Climate change will lead to severe reductions of distribution area of the genetically diverse Zambezian (− 41–− 54%) and Southern (− 63–− 82%) phylogroups, but to an increase for the genetically depauperate Sudano-Sahelian (+ 7– + 26%) phylogroups. This study improves our understanding of the impact of climate change on the future distribution of this species. This knowledge is particularly useful for biodiversity management as the conservation of genetic resources needs to be considered in complementary strategies of in-situ conservation and assisted migration.

## Introduction

Contemporary patterns of genetic variation have become a crucial aspect of research in biogeography, conservation and evolutionary biology^1–3^. Major drivers of intraspecific genetic variation include climatic shifts during the Quaternary, ecological gradients, demographic processes and anthropogenic land-use change^4–6^. Changes in geographic ranges driven by fluctuations in climate have fragmented or reconnected populations of the same species^7^. This can lead to spatial structuring of genetic diversity in distinct gene pools^2^ where a phylogeographic discontinuity is interpreted as evidence for range fragmentation during past climatic changes^8^.

Recent advances in phylogeography and landscape genetics have focused on identifying and understanding genetic structure, including the influence of geographic and environmental heterogeneity, on spatial genetic variation with possible implications for forecasting the distribution of gene pools under climate change^3,9,10^. There is sufficient evidence that modern climate change is reshuffling the geographic distributions of plant and animal species world-wide^11,12^. Populations or species react to climate change by either persisting, dispersing to suitable ecological conditions, or going extinct, which influences the amount and distribution of intraspecific genetic diversity^13^. Despite the increasing evidence for global climate warming, model predictions of how climate will change in the course of the twenty-first century is treated with some level of uncertainty^13,14^. This is addressed by assuming that emissions will follow one of several scenarios known as the Representative Concentration Pathways (RCPs)^15–17^. Global climate change projections show a strong warming trend over the twenty-first century and Africa has been identified as one of the regions of the world most vulnerable to climate change^18^. Particularly in sub-Saharan Africa, temperature is expected to rise by approximately + 2.0 to + 4.5 °C by 2100^19,20^.

The savannah belt of sub-Saharan Africa is affected by significant climatic, ecological and geological barriers^21,22^, resulting in major partitions into the Sudano-Sahelian (West—Central Africa), Zambezian (East Africa) and Southern African biogeographic regions^23^ for both plant and animal species^21,22^. Prominent historical barriers include the Mega Lake Chad, Dahomey Gap, East African Rift valley, Ethiopian highlands, Adamawa Highlands, Namib desert and Kalahari desert^21,22,24,25^, hindering dispersal and gene flow among regions. Documented evidence shows that tropical savannah and woodland trees potentially played an important role in forest assemblages during the Last Glacial Maxumum (LGM)^22^. Phylogeographic studies of these tree species have been instrumental in unravelling the role of past environmental changes in the structuring of genetic diversity found in contemporary populations^22^. Recently, new insights into the geographic distribution and range dynamics of many African savannah tree species have been provided by the analyses of ecological data, while additional insights come from analysing the pattern and distribution of genetic diversity within species^8,22,26,27^. In some species, intraspecific phylogeographic patterns mirror biogeographic patterns. For example, *Prunus africana* (Hook.f.) Kalkmanis genetically structures into Sudanian, Zambezian and Southern gene pools^26^, while genetic discontinuities in the form of parapatric genetic clusters were detected in the Sudanian savannah for *Vitellaria paradoxa* C.F. Gaertner and *Parkia biglobosa* (Jacq.) R.Br ex G. Don^28,29^. However, the absence of clear-cut genetic discontinuities over large distances has been reported for other species, including *Adansonia digitata* Linn.^30^, *Khaya senegalensis* (Desr.) A. Juss.^22^, *Afzelia africana* Sm. ex Pers. in the Sudanian, and *Afzelia quanzensis* Welw. in the Zambezian region^8^. Although some of these studies investigated the impact of historical perturbations on genetic patterns^31^, there is a lack of studies into the genetic consequences of climate change for the future distribution of species and their intraspecific groups.

**Figure 1:**
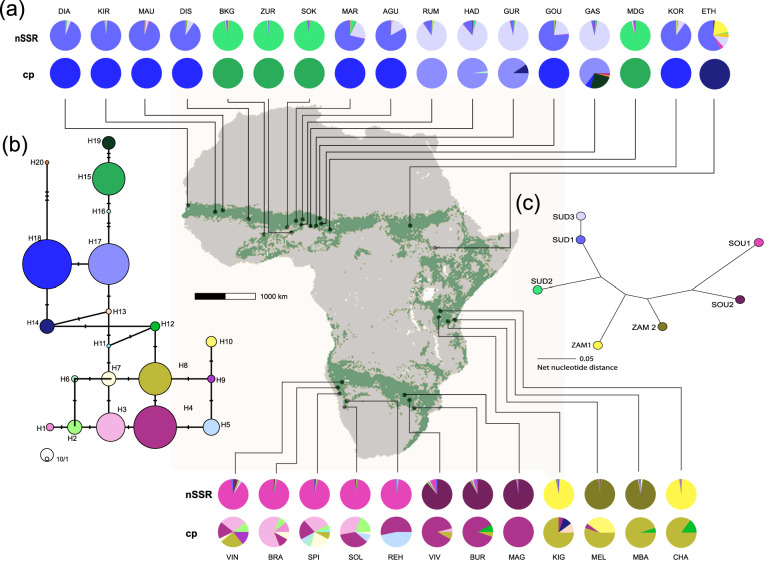
(**a**) The Geographic occurrence of populations of *Senegalia senegal* analyzed, showing the proportional assignment to both, seven gene pools of nSSR and cpDNA haplotypes. Each pie chart represents one population and the colors within the pie chart depict the distinctive haplotypes/gene pools. (**b**) Median‐joining network of the 20 cpDNA haplotypes (H1-H20). Circle sizes are proportional to haplotype frequencies. Small bars indicate the number of mutational steps in case more than one step occurred. (**c**) Neighbor-joining tree showing the relationship among seven nSSR gene pools (*K* = 7) as revealed by STRUCTURE (Fig. 2). The map was downloaded from WORLDCLIM^41^ and modified manually. The green area in the map background indicates the modeled potential distribution of *S. senegal*^27^ generated using ArcGIS Desktop ver. 10.5^42^. See Supplementary Table S1 for population details.

Here, we assess the impact of climate change on intraspecific lineages of a savannah taxon using *S. senegal* (L.) Britton (Fabaceae, Mimosoideae), commonly known as gum arabic, one of the most important tree species in the tropical woodlands of sub-Saharan Africa both economically and ecologically^32^. *Senegalia senegal* has an extensive geographic coverage on the African continent and is common to habitats that experienced past range contraction and expansions due to Pleistocene climatic fluctuation (Fig. 1)^33,34^, and has ecological attributes such as resilience to adverse environmental conditions^35,36^. The species is distributed throughout the African arid and semi-arid regions, extending from Senegal along the Sudano-Sahelian zone to the Red Sea and then southwards through the dry savannah and montane areas of the Zambezian region into southern Africa^37^. A phylogeographic study on *S. senegal* based on nuclear and plastid genome data has suggested an evolutionary origin in East or Southern Africa and reported a major division separating eastern and southern African populations from those in West and Central Africa, suggesting a recent range expansion starting from East Africa^38^. Historical distribution and range dynamics of *S. senegal* indicate variation along climatic and edaphic regimes, separating the eastern and southern ranges from the western and central African ranges^27^. In addition, population genetic studies of Kenyan and West African populations of *S. senegal* have shown that anthropogenic perturbations and climatic shifts could impact levels of genetic diversity (GD), population sizes, structure of gene pools, and natural regeneration patterns of the species at regional scale^6,39^. Investigating the impact of climate change on GD of *S. senegal* will provide insights into population structure, and can further advance our understanding of the genetic consequences of post-glacial expansion processes and climate warming on the future distribution of genetic variation. We here use a comprehensive data set of nuclear and chloroplast microsatellite data and combine phylogeography, landscape genetics and distribution range modeling to investigate how future climate change will affect the distribution of phylogeographic groups (hereafter referred to as phylogroups) and their gene pools across Africa. Specifically, we address the following questions:

1. How is phylogeographic structure reflected in levels of genetic variation within populations? Population expansion often leads to the loss of genetic variation due to genetic drift or bottleneck effects. We thus hypothesize that within-population GD declines with distance from the proposed origin of the range expansion towards West Africa^40^.
2. How do environmental conditions in the local habitat of *S. senegal* correlate with genetic differentiation among populations? Landscape characteristics (e.g., barriers to migration), past range changes and habitat fragmentation can influence population genetic structure^3,10^, and heterogeneity in landscape features has been shown to impact genetic variation in east African populations of *Senegalia mellifera*^24^. Thus, the highly contrasting ecological conditions across sub-Saharan Africa are expected to be reflected in patterns of genetic variation. We hypothesize that population genetic differentiation is correlated with geographic and environmental distance resulting in both patterns of isolation by distance (IBD) and isolation by environment (IBE).
3. What are the genetic consequences of future climate change for *S. senegal*? Climatic changes may lead to conditions that fall outside the current environmental tolerances of populations and may trigger either a geographic range shift, ecological adaptation or extinction^13,40^. Assuming niche conservatism (i.e., populations are unable to tolerate the new conditions locally, e.g., through phenotypic plasticity or adaptation), populations will need to track their current environmental tolerances by geographic range shift. Depending on the overall magnitude, direction and rate of climate change, species may suffer potentially severe reduction of suitable habitat and in turn loss of population GD. We predict that the ongoing climate change will lead to a loss of GD due to an overall reduction of range size. We use a population genetic approach, with an improved marker resolution and larger sample size compared to previous attempts^38^ to refine the phylogeographic structure of African *S. senegal*. In addition, we model the potential impact of climate change on the distribution of phylogeographic groups of *S. senegal* using SDMs. This study will increase our understanding of the spatial structuring of genetic variation including how phylogeographic groups may respond under future environmental change scenarios.

## Results

### Haplotype distribution and phylogeographic patterns

Two chloroplast (cpSSR) loci yielded 20 haplotypes across the 29 populations from which samples of *S. senegal* were collected (Fig. 1). Between one and eight haplotypes were found per population. In the Sudano-Sahelian range, except for three populations (HAD, GUR and GAS), all other populations were fixed to a single haplotype. Contrastingly, in both the Zambezian and Southern ranges, almost all populations harboured more than one haplotype.

A strong phylogeographic division between the Zambezian-Southern ranges (haplotypes H1-H11) and the Sudano-Sahelian range (haplotypes H15-H20) is visible in the haplotype network (Fig. 1a). Six mutational steps separate these haplotype groups and they are connected by four intermediate haplotypes (H11-H14) (Fig. 1). The six most common haplotypes indicate different regions they dominate, however, without being restricted to them: in the Zambesian-Southern range, H3 dominated in the Namib, H4 in Kalahari, and H8 in the Zambesian range, whereas for the Sudano-Sahelian group, H17 + H18 dominated in the Sahelian and H15 in the Sudanian region. The genetically intermediate haplotypes either occurred in geographically intermediate populations, like H14, which is dominant in the easternmost Sudano-Sahelian site ETH connecting the Sudano-Sahelian and Zambezian ranges, or intermediate haplotypes were shared between ranges, like H12 which is shared between the Zambezian and Southern range.

As expected for non-recombining plastid markers, population differentiation was very strong (Supplementary Table 2). A non-hierarchical AMOVA for cpSSR showed 73% genetic variation among populations (Ф_PT_ = 0.733), whereas hierarchical AMOVAs, using the STRUCTURE clusters as groups (see below) found increasing variance among clusters when more clusters were considered, starting at 43% (Ф_RT_ = 0.427) at *K* = 2, and 48% (Ф_RT_ = 0.485) at *K* = 3, reaching 90% (Ф_RT_ = 0.901) at *K* = 7 (Supplementary Table 2).

### Genetic population structure

**Figure 2:**
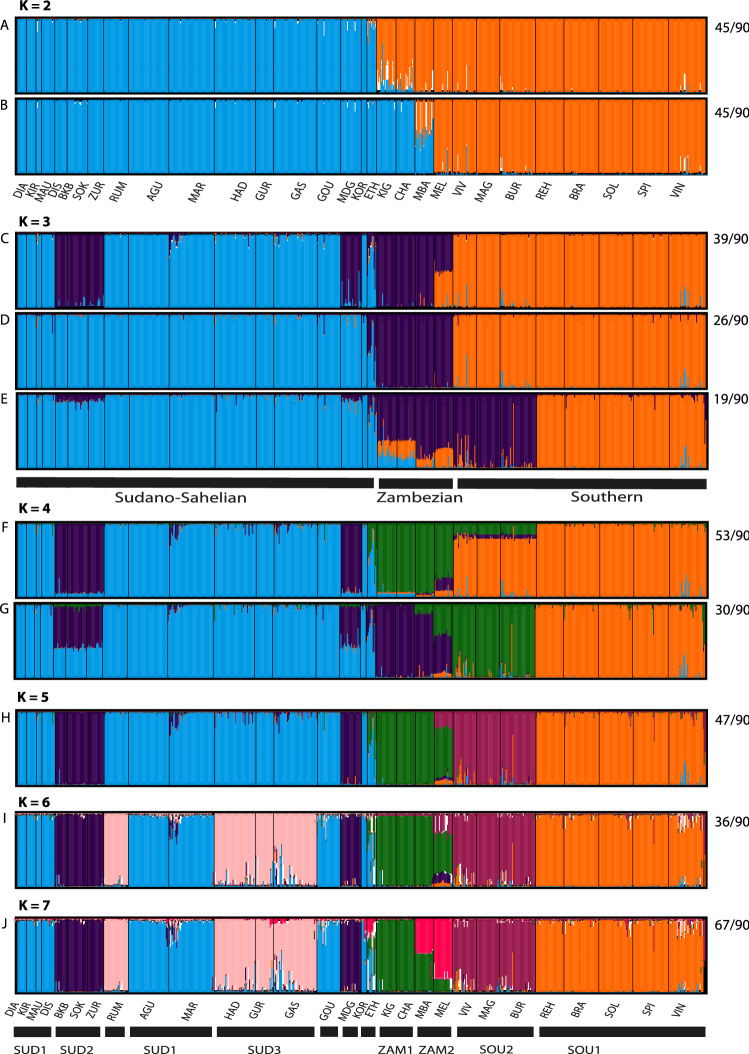
Bar plots of individual proportional affiliation to genetic clusters identified by STRUCTURE for the 730 individuals genotyped at ten nSSR and two cpSSR loci for *K* = 2–7. For *K* = 2–4 major and minor modes are shown as identified by clumpak^43^, while for *K* = 5–7 only the major mode is shown for simplicity. The frequency of modes among 90 runs is given to the right. Vertical bars represent individual samples. See Fig. 1 for geographic location of sites.

Bayesian analysis of population structure of the combined nuclear and chloroplast data sets yielded a hierarchical pattern across the African range (Fig. 2). The uppermost hierarchical levels were two gene pools as suggested by the method of Evanno et al. (2008) (Supplementary Fig. S1). However, while one pool represented the Sudano-Sahelian region and the other Southern Africa, the Zambezian region grouped equally likely with either of them in two different clustering modes (Fig. 2A,B). The likelihood of data strongly increased for values of *K* > 2 with (Supplementary Fig. S1) and new emerging clusters generally encompassed whole populations clearly indicating additional biologically relevant and geographically coherent hierarchical genetic structure. At *K* = 3, three gene pools coincided largely with the Sudano-Sahelian, Zambezian and Southern African biogeographic regions. However, the cluster of the Zambezian range encompassed either only populations from East Africa, or included a few populations from either West- or South-Africa in three different modal solutions (Fig. 2C–E). In all runs at *K* = 3 the third cluster included all populations of the Zambesian biogeographic range. At *K* = 4, two contrasting patterns are found in two modes which are resolved at *K* = 5, where the former Sudano-Sahelian cluster separated into the Sahelian (light blue) and Sudanian (dark blue) biogeographic regions and the Southern African region separated into Namib (SOU1) and Kalahari (SOU2). At *K* = 6, the Sudano-Sahelian range was further divided in the western-most (SUD1, light blue), the southern (SUD2, dark blue) and the central (SUD3, pink) populations. At *K* = 7, the Zambesian range split into two gene pools, representing north-western (ZAM1) and south-eastern (ZAM2) populations. At *K* = 7, the major mode comprised 67/90 (74%) of the STRUCTURE runs (Fig. 2J), which was the highest proportion observed across all levels of *K*, strongly suggesting that *K* = 7 can be considered as both comprehensive and consistent.

With the nuclear data set, non-hierarchical AMOVA revealed 29% variation among populations (*F*_ST_ = 0.287; P ≤ 0.001). Hierarchical AMOVAs revealed increasing variation among clusters with increasing *K*, ranging from 17% (*F*_RT_ = 0.171) at *K* = 2, 20% (*F*_RT_ = 0.201) at *K* = 3 up to 25% (*F*_RT_ = 0.247; Supplementary Table 2) at K = *7*.

### Genetic variation within populations

**Figure 3:**
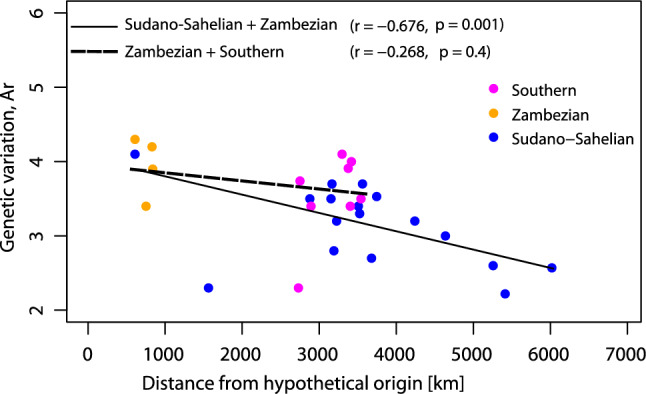
Genetic variation (rarified allelic richness, Ar) of populations of *Senegalia senegal* as a function of distance from assumed East African origin of the range expansion into the Sudano-Sahelian region and between Eastern and Southern ranges only.

Our results show high genetic variation for *S. senegal*, which is strongly structured across the species’ range in Africa (Fig. 1). Genetic diversity at the population level as indicated by allelic richness (A_r_) significantly declined with distance from the assumed East African origin of the range expansion into West Africa (r = − 0.676; r^2^ = 0.457; *p* = 0.001, Fig. 3, Table S3). In contrast, genetic variation did not decline between the Zambezian and the Southern subrange (r = − 0.268, *p* = 0.4). This pattern is consistent with range expansion from East to West with accompanying bottlenecks and genetic drift, while this was not the case for the Eastern and Southern ranges. The sub-range means for nuclear markers (nSSR, Table 1) show that A_r_ is significantly lower in the Sudano-Sahelian in comparison to the Zambezian and Southern regions, while A_priv_ is higher in the Zambezian than Sudano-Sahelian and Southern regions, and *F*_is_ is higher in the Southern than Sudano-Sahelian and Zambezian regions. Interestingly, sub-range estimates for plastid markers (cpSSR) indicate that the diversity of haplotypes was significantly higher in the Southern range, with a total of 11 haplotypes present compared to eight and six in the Sudano-Sahelian and Zambezian ranges (Table 1, Table S3).

**Table 1:** Sub-range genetic diversity estimates at ten nuclear SSR loci and two cpSSR loci for *Senegalia senegal* in three biogeographic regions*.* Given are the mean values across populations (with different letters indicating significant differences according to ANOVA and posthoc test) and region estimates in which regions are treated as populations. See Supplemental Table S3 for population level estimates.

	nSSR							cpSSR					
Region	N	A_r_	Na	A_priv_	*H*_*o*_	*H*_*e*_	*F*_is_	A	P	Ne	Rh	Dv	D^2^_sh_
**Mean values across populations within region**													
Sudano-Sahelian	22.4	3.14b	4.1b	1.24b	0.62a	0.56a	− 0.12b	1.35b	0.18a	1.08b	0.12b	0.05b	0.38a
Zambezian	23.0	3.95a	5.93a	10a	0.62a	0.62a	0.0008ab	2.75ab	0.5a	1.58ab	0.90ab	0.34a	0.64a
Southern	23.6	3.54ab	6.38a	2.75b	0.42b	0.54a	0.20a	4.13a	0.5a	2.7a	1.55a	0.50a	0.62a
**Region estimates**													
Sudano-Sahelian	374	9.11	12.4	33	0.579	0.626	0.075	8	6	3.135	4.488	0.683	3.163
Zambezian	80.9	13.48	13.5	54	0.588	0.763	0.23	6	2	1.616	5.000	0.386	0.824
Southern	267.1	9.84	13	46	0.409	0.646	0.368	11	8	3.536	8.146	0.720	1.094

N, number of samples per location; A_r,_ rarified allelic richness (Mousadik and Petit, 1996); Na, mean number of alleles per locus per population; *H*_*o*_, observed heterozygosity; *H*_*e*_, expected heterozygosity; A_priv_, number of private alleles; *F*_is_, inbreeding coefficient; A, number of haplotypes; P, number of private haplotypes; Ne, effective number of haplotypes; Rh, haplotypic richness; Dv, genetic diversity; D^2^_sh_, mean genetic distance between individuals.

### Geographic and environmental drivers of genetic structure

**Figure 4:**
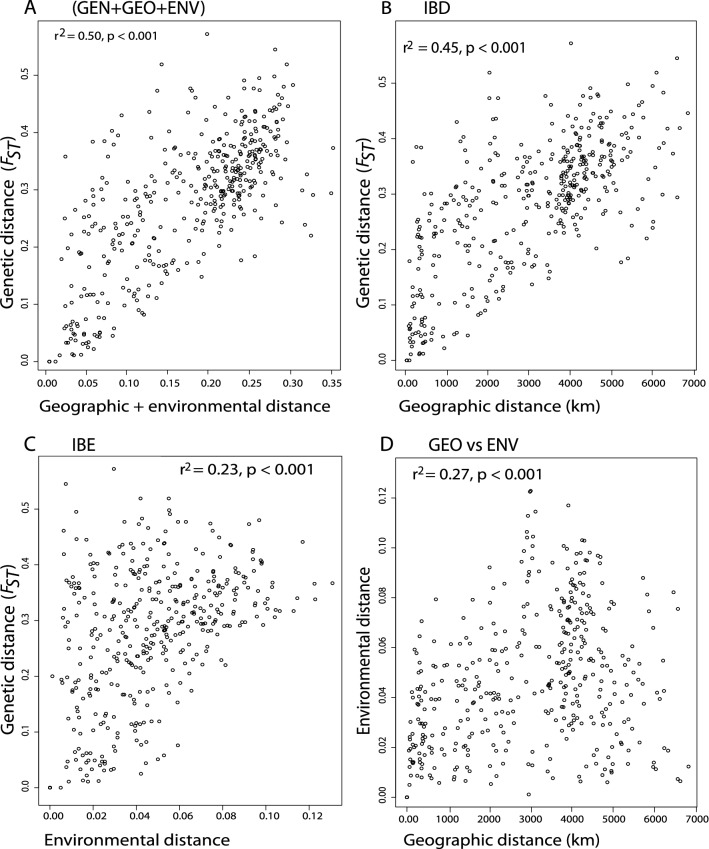
Multiple matrix regression with randomization analysis on *Senegalia senegal* in Africa. Scatterplots show patterns of (**A**) the combined effects of geographic and environmental distances on genetic distance; (**B**) Isolation by distance; (**C**) Isolation by environment; (**D**) the relationship between geographic and environmental distances.

The range-wide MMRR analysis shows that geographic and environmental distances were both associated with genetic distances (Fig. 4; Table 2). Geography and environment jointly explained 50% (r^2^ = 0.50, *p* < 0.001) of variation (Fig. 4a) with geography accounting for the largest proportion of variation (45%) in genetic distance (Fig. 4b). The signal for IBE was driven by the precipitation of the wettest month (Bio13), which had a significant association with genetic distances in contrast to the other climatic and soil variables considered in the analysis (Fig. 4c, Table 2). The analysis also revealed a weak but significant correlation between geographic and environmental distances (r^2^ = 0.267, *p* < 0.001, Fig. 4d) with Bio13 and monthly variability in potential evapotranspiration (PETseasonality) as the most important.

**Table 2:** Multiple matrix regression with randomization (MMRR) model testing for range wide isolation by distance and isolation by environment for *Senegalia senegal.*

Variable distance	Coefficient	T-statistic	p-value
Geographic distance	4.12E-05	14.671	0.001
Bio13	4.52E-04	4.617	0.006
PETseasonality	2.23E-08	3.723	0.067
Soil pH	− 8.20E-04	− 0.803	0.608
Bio8	− 6.12E-05	− 0.237	0.888
Intercept	0.111	10.624	1

When the analyses were repeated at the subrange level, significant IBD was detected for the combined Zambezian and Southern ranges (r^2^ = 0.38) and for the combined Sudano-Sahelian and Zambezian subrange (r^2^ = 0.29), pointing to an older or more established relationship in Zambezian—Southern than in the Sudano-Sahelian—Zambezian ranges (Supplementary Fig. 2). Accordingly, IBE was detected in Sudano-Sahelian—Zambezian range (r^2^ = 0.32, *p* = 0.001) but not significant in the Zambezian—Southern (Supplementary Fig. 2). Environmental distance was partly correlated to geographic distance in the Sudano-Sahelian—Zambezian (r^2^ = 0.43; *p* = 0.001). However, our data did not show a significant correlation in the Zambezian—Southern range indicating that both parameters are acting in concert with the other in the Sudanian—Zambezian range but maybe independent in the Zambezian—Southern range (Supplementary Fig. 2).

### Habitat suitability modeling

**Figure 5:**
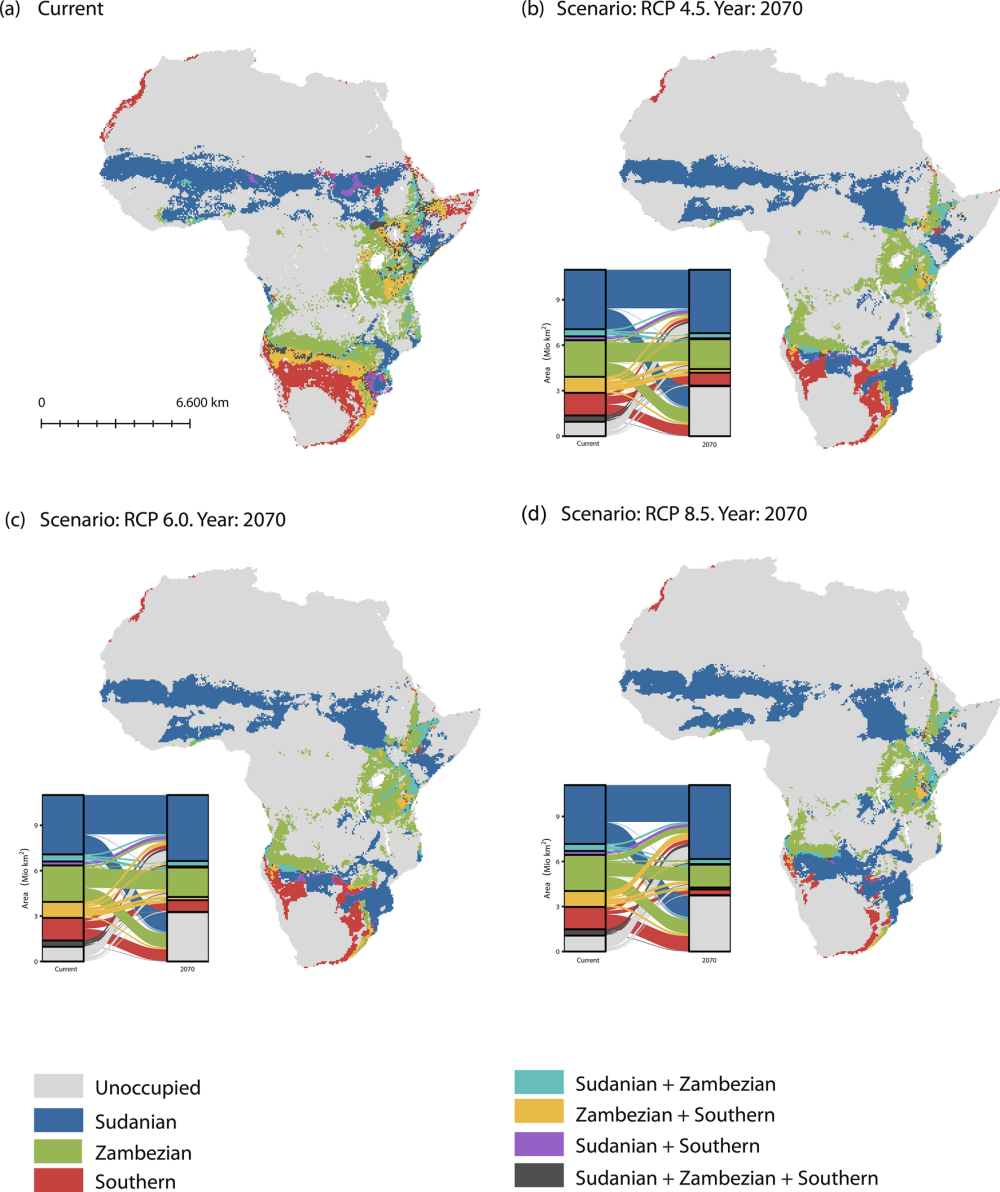
Potential current and future (2070) distribution of three phylogeographic groups of *Senegalia senegal* (Sudano-Sahelian, Zambezian and Southern) in Africa according to Biomod2 distribution modelling. (**a**) Present-day suitability; (**b**) intermediate emission RCP 4.5 scenario; (**c**) high emission RCP 6.0 scenario and (**d**) very high emission RCP 8.5 scenario projected with CCSM4 model. Inset (**b**–**d**) are Sankey plots showing how the area occupied by the phylogroups will change for three climate change scenarios. The shape files for the maps were downloaded from WORLDCLIM^41^. The modeled potential future distribution of *S. senegal* were generated using ArcGIS Desktop ver. 10.5^42^ .

The habitat suitability model results for future projections by 2070 are largely congruent for the CCSM4 and MIROC5 scenarios and only results for CCSM4 are presented (Fig. 5, Table 3). Climate‐based distribution models of *S. senegal*, built with current conditions, generally indicated the presence of a continuous potential distribution for the species throughout its known distribution range in sub-Saharan Africa (Fig. 5a). The accuracy values obtained for the six ensemble methods indicated a good performance and agreement of the model to the data (Supplementary Table 3). Across the three phylogroups, a decrease of the total suitable area between 22.8% (RCP 6.0) and 26.6% (RCP 8.5) is predicted (Table 3, Fig. 5). However, the moderate reduction of the total range is accompanied by a drastic change of the share and distribution of the three phylogroups. Depending on the scenario, the future total range for the Sudano-Sahelian group may decline by up to 8.4% (RCP 4.5) or increase up to 5.1% (RCP 8.5). However, the area where the Sudano-Sahelian group does not overlap with other groups is predicted to increase for all scenarios by between 6.7% (RCP 4.5) and 25.6% (RCP 8.5). Large parts of this gain of suitable area is located in the Zambezian and Southern range. In contrast, the Zambezian group is predicted to lose about half of its total range (40.7–53.7%), with a loss between 18.3% (RCP 4.5) and 37% (RCP 8.5) predicted for the Zambesian-only areas. Most drastic reductions of suitable area are predicted for the Southern range, declining between 63% (RCP 4.5) and 82% (RCP 8.5) of the total area and between 42.8% (RCP 4.5) and 74.6% (RCP 8.5) considering the Southern-only area. While in general the zones of geographic overlap of the ranges of the different phylogroups are of minor importance, a considerable area of overlap was found between the Zambezian and Southern phylogroup for the potential current distribution, which strongly declined in all future scenarios.

**Table 3:** Current suitable area and predicted area (as percentage of current area) for each phylogroups of *Senegalia senegal* and their co-occurrence for the three climate change scenarios RCP 4.5, RCP 6.0 and RCP 8.5 for the year 2070. Predicted area percentage > 100% and < 100% indicates gain and loss of area, respectively.

Phylogroup	Current Area (km^2^)	Percentage (%) of current area for 2070		
		RCP 4.5	RCP 6.0	RCP 8.5
Total range	1.17 × 10^7^	76.6	77.2	73.4
Sudano-Sahelian Total	5.89 × 10^6^	91.6	95.0	105.1
Zambezian Total	5.0 × 10^6^	59.3	58.4	46.3
Southern Total	3.76 × 10^6^	37.0	33.5	17.9
Sudano-Sahelian only	4.56 × 10^6^	106.7	111.0	125.6
Zambezian only	2.80 × 10^6^	81.7	80.7	63.0
Southern only	1.73 × 10^6^	57.2	51.5	25.4
Sudano-Sahelian + Zambezian	5.43 × 10^5^	71.8	77.0	72.4
Sudano-Sahelian + Southern	3.03 × 10^5^	24.8	28.6	14.0
Zambezian + Southern	1.22 × 10^6^	22.0	20.4	12.4
Sudano-Sahelian + Zambezian + Southern	4.87 × 10^5^	9.9	5.8	6.1

## Discussion

In this study, we used molecular markers to highlight the phylogeography and population-level genetic diversity in the African distribution range of *S. senegal*. Three major phylogeographic groups were identified in Eastern, Southern and Western Africa. Furthermore, our results provide support for both isolation by distance (IBD) and isolation by environment (IBE) in the genetic structuring of *S. senegal.* Our SDM projections predict different impacts of climate change on the distribution of the phylogeographic groups under future environmental change scenarios with evolutionary older groups being most drastically affected.

Range-wide heterozygosity levels obtained from this study (*H*_e_ = 0.56) are very similar to levels obtained in previous analyses of microsatellite markers of *S. senegal* populations in western Africa (*H*_e_ = 0.54–0.56)^44^ but slightly lower than in the eastern range in Kenya (*H*_e_ = 0.617)^6^. This may be due to the use of slightly different sets of markers and differences in sample size. Within our study, genetic diversity estimates were highest in the Zambezian and the Southern ranges, the latter showing highest diversity of plastid markers among the subranges. This finding corroborates earlier analyses finding higher diversity in eastern^6^ and in southern Africa^38^ compared to Sudano-Sahelian populations in Western Africa. Genetic diversity in Southern Africa may even be underestimated as indicated by the presence of null-alleles in that region. A distance-dependent decline of diversity from Eastern to Western Africa indicates evolutionarily younger populations are affected by, e.g., bottlenecks and genetic drift during range expansion from East to West Africa^38^. In addition, increasingly unfavorable climatic conditions may have contributed to drift as indicated by the observed IBE. A similar pattern of an East-to-West decline of genetic diversity across Africa was found in *P. africana*^26^. However, contrasting patterns with higher diversity in the Sudanian are known for other tree species^29,31,45^. Thus, the relationships found across sub Saharan Africa corroborate a general pattern of diversity clines that are correlated with climate driven range changes, as e.g. observed in the Mediterranean^46^.

### Population genetic structure and phylogeographic patterns

Although *S. senegal* currently has a continuous distribution from west (Senegal) to east (Ethiopia) sub-Saharan Africa^27^, our data show a strong genetic separation between West Africa, i.e. the Sudano-Sahelian biogeographic regions, and East and Southern Africa, i.e. Zambesian and Southern African biogeographic regions. Molecular data were largely congruent between nuclear and plastid genomes at the range wide scale, both showing similar regional genetic structuring and phylogeographic patterns.

Our population level GD data are in line with phylogenetic evidence of a sister group relationship between West- and East-Africa^38^, and thus corroborate an evolutionary origin of the West African *S. senegal* in the eastern region. The scenario of colonization from East to West Africa is supported by the relatively small genetic distances (< 4 mutations) from the two dominant West African haplotypes (H17, H18), suggesting that these haplotypes have originated from H8 in the East. Assuming relatively stable environmental conditions during the time frame of range expansion, dispersal from East Africa likely has occurred via Ethiopia-Sudan-northeast Nigeria and westwards to the rest of the Sudano-Sahelian distribution range.

Overall, population differentiation was high at both chloroplast (Ф_PT_ = 0.733) and nuclear markers (*F*_ST_ = 0.287) and higher than previous values for regional assessments^39,44,47^ due to differentiation among phylogroups at the range level, in particular as we included the Namib region in our analysis (Table 1, Supplementary Table 2). However, within regions, we found similar values of differentiation as previously reported for western (0.145)^39^ and eastern (0.045)^47^ Africa respectively. The *K* = 3 partition in STRUCTURE largely reflected the phylogeographic regions of Sudano-Sahelian, Zambezian and Southern ranges. Pairwise *F*_ST_ revealed that the highest differentiations occur between populations from Sudano-Sahelian and Southern ranges, consistent with a range-wide pattern of isolation by distance. Population genetic differentiation can result from evolutionary processes of mutation and genetic drift, demography, anthropogenic disturbances, geographic isolation and limited or restricted gene flow among populations^5,6,48^. The high GD coupled with endemic gene pools in the Southern range may be due to barriers to gene flow, triggered by the heterogeneous complex mosaic of landscapes^38^ among the surrounding populations. In addition to demographic history^49^, spatially heterogeneous landscapes in most cases support greater genetic and species diversity^50–52^. The presence of mountains (e.g., Spitzkoppe, Erongo region and Brandberg, Naukluft) and inhospitable landscapes (e.g., Khormas highlands that seperates populations Rehoboth and Solitaire) within the sampled range in Namibia, might be acting as a barrier, thus limiting gene flow and facilitating differentiation in local populations. Greater genetic diversity increases the likelihood that appropriate adaptive variation will be available to facilitate adaptation to the new conditions^52,53^. Our results suggest gene flow limitation between regions resulting in considerable genetic differentiation among phylogeographic lineages. However, populations have remained connected within phylogroups throughout the large, continuous Sudano-Sahelian or Zambezian savannahs.

In deciphering the role of geography and environment as drivers of genetic structure, we found strong relationships between genetic differentiation and both geographic distance (IBD) and environmental distance (IBE), with IBD explaining the majority of genetic differentiation and IBE also contributing significantly. The IBD model assumes that genetic differentiation increases among populations with geographic distance due to limited gene flow and drift^54^. The strong pattern of geographic isolation observed in our dataset can be explained by either geographic distance, or landscape barriers (e.g., Lake Chad, along the Sudano-Sahelian biogeographic region and the Rift valley in eastern Africa) between populations of *S. senegal*. IBD patterns are commonly found in large scale analyses of widespread plant species^8,55^ unless either gene flow or drift are dominating^56,57^. Isolation by environment (IBE) patterns are caused by environmental heterogeneity and local adaptation related to strong divergent selection^58,59^. Although IBE patterns for *S. senegal* were generally weak on a range-wide scale, slightly stronger ecological isolation observed at the subrange level could be explained by either difference in the occupied environmental space, i.e., regional environmental space differentiation^27^, or selection and local adaptation^59–61^. Thus, populations that might have dispersed to other suitable ranges within Africa over time adapted to the prevailing local conditions through selection. This result suggests that IBE in *S. senegal* is primarily driven by differences in temperature during the wettest months in the species local habitat. In addition, different flowering times were observed among population during fieldwork to collect samples. It should be noted that differences in temperature regimes between populations might cause differences in phenology, with reduced overlap of flowering time potentially leading to partial reproductive isolation^62^. This pattern of reduced overlap in reproductive timing known as isolation by time^63^ may additionally be a contributing factor driving genetic structure in *S. senegal*.

By analyzing changes in the realized environment of *S. senegal*, we quantified and mapped declining and expanding phylogroups that are projected as a result of the twenty-first century climate change. In principle, the projections across the three future climate scenarios present a complementary pattern of range gain for the Sudano-Sahelian phylogroup and range loss for the Zambezian and Southern phylogroups (Fig. 5). As the rate of loss scales with temperature increase, the ultimate extent of climate change will be critical, especially for the Southern populations of *S. senegal.* The zones of current geographic overlap between the three phylogroups (e.g., in Sudan-Ethiopia and southern Angola), represent areas where highest levels of genetic variation are to be expected. Anthropogenic climate warming is projected to cause the disappearance of phylogroups in these regions of overlap for the next 50-plus years across all scenarios considered in this study, consequently leading to the loss of GD (Fig. 5). Increase in the mean annual temperature by 1.1 to 6.4 degrees Celsius within the twenty-first century^18,64^ may cause previously well-adapted genotypes to lose their competitive advantage, by selection favouring other genotypes already present in the population or newly immigrating genetic variants^65^. In general, whether local population will go extinct will also depend on the presence of phenotypic plasticity and its genetic basis and on small scale opportunities for suitable environmental conditions within the existing range.

The highly genetically diverse Southern populations of *S. senegal* are predicted to be strongly reduced in extent and go regionally extinct in the future. In contrast, genetically depauperate populations in the Sudano-Sahelian range might not be at high risk of extinction, as they may exist in areas of predicted suitable climate both inside and outside of their currently occupied range with a potential for range expansion (Table 3, Fig. 5). However, our results are valid within the framework of range suitability modeling and do not explicitly consider the dispersal rate for *S. senegal*. Thus, future suitable area distant from the current range may actually be out of reach by natural means, unless human-assisted migration is considered (see below). Generally, our results show evidence for severe future range loss for the evolutionary older populations (Zambezian and Southern), with a negative impact on unique genetic variation of these phylogroups in comparison to the evolutionary younger and genetically depauperate Sudano-Sahelian phylogroup^11^. The mid-portion of the currently occupied native range of the Southern phylogroup might be lost to the expanding Sudano-Sahelian phylogroup during the intermediate scenarios and even more severe range retraction during the RCP 8.5 scenario, leaving extant populations as fragments in the Namib region as well as in the Mediterranean scrub of the south-eastern tip of Africa. The extremely localized high-elevation populations that harbour highest levels of genetic diversity within the whole species range occur in the Namib region (Spitzkoppe and Brandberg mountains) and might be at risk. Overall, a range reduction of the Zambezian and Southern phylogroup and potential range extension of the Sudano-Sahelian phylogroup would result in a movement from North to South, in contrast to a South to North movement observed in the northern hemisphere^66^.

### Implications for conservation

There is growing concern over the global rate of environmental change. This situation has raised further concerns about whether organisms, especially plant species can keep track, by migration or evolution, with the predicted changing distribution and spatial arrangement of suitable habitat^67,68^. Our model highlights that the Zambesian and Southern phylogroups of *S. senegal* will be at risk due to climate change. Three options may allow them to contend with rapidly changing environments: dispersal, phenotypic plasticity, or adaptation^11,69,70^. Although *S. senegal* is known to exhibit potential for long-distance dispersal^32,38^, it is uncertain whether phylogroups, e.g., populations from the Sudano-Sahelian phylogroup might be able to track a suitable habitat, predicted to occur outside of its currently occupied range in the far Southern range. However, high levels of GD observed for *S. senegal* in this study might ultimately determine the fate of the species through a rapid adaptive change should, for example, the species be incapable of a plastic response. Greater GD increases the likelihood that appropriate adaptive variation will be available for adaptation to the new conditions. Evolutionary adaptation, however, will need time, and large and reproductively active populations. Therefore, regions with highly diverse extant gene pools predicted to be extirpated or go extinct in the future (e.g. Southern phylogroup), should be high priority areas for both in situ and ex situ conservation. Our model predicted largely non-overlapping distribution areas for the phylogroups based on environmental suitability and thus may guide assisted migration programs. However, while our modeling provides predictions on where the phylgroups would find suitable climate in the future, we propose that common gardens should first be established across different biogeographic regions to prove adaptedness prior to the implementation of assisted migration using the most appropriate seed material^71,72^.

Additionally, forecasting large-scale species distributions is becoming a crucial component for conservation planning, especially for ecological and commercially relevant species^73^. If the projected range loss of *S. senegal* leads to fragmentation, then these changes have severe implications for the species through genetic drift, gene flow and inbreeding depression^74,75^. This will require enhanced conservation efforts, including proactive and intensive management, to provide greater flexibility for the species to respond successfully and avoid extinction. We hope that our study will provide a clear directive on the genetic consequences of climate change on this economically and ecologically important savannah tree species and that the understanding from the findings will support the development or re-designing of effective conservation strategies in *S. senegal,* in particular considering intraspecific genetic groups and their individual predicted fate. Furthermore, several other tree species have similarly wide distributions across the drier parts of Africa^26,76,77^. Therefore, it would be interesting to investigate whether the phylogeographic patterns as well as the potential impact of future climate change observed for *S. senegal* can serve as a model for other tree species occupying the savannah type environments. Finally, we hope our study will be of interest to biodiversity stakeholders and can be integrated in the conservation programs of the United Nations Environment Programme (UNEP), United Nations Convention to Combat Desertification (UNCCD), Intergovernmental Science-Policy Platform on Biodiversity and Ecosystem Services (IPBES), National Biodiversity Strategy and Action Plan (NBSAP) and important plant areas (IPAs).

## Methods

### Sampling strategy

The African range of *S. senegal* encompasses tropical woodland, open savannah and semi-desert steppe, encompassing biogeographically the Sudano-Sahelian region that extends from extreme western Africa to the Zambezian region in eastern Africa and then southwards up-to the Kalahari and Namib regions^21^. We did not consider the small Asian part of the species range. Fresh leaf samples were collected from 746 individuals of *S. senegal* across 29 locations covering most of its geographic range in Africa (Fig. 1, Supplementary Table 1). We aimed at 20 samples per site, but included populations with > 5 samples, resulting in an average of 25 samples, but correcting for biased sample size by rarefaction (see below). A distance of 10 m was maintained between sampled individuals within populations. Field-collected material was dried in silica gel before DNA extraction. At least one individual per population was deposited as a voucher specimen at the herbaria of the National Centre for Genetic Resources and Biotechnology (NACGRAB), Ibadan, Nigeria, at Leipzig University herbarium (LZ), Germany, and the National Botanical Research Institute, Windhoek, Namibia (Supplementary Table 7).

### Genetic variation and population structure

We used ten diploid nuclear simple sequence repeat (nSSR), i.e. biparentally inherited, markers developed for *S. senegal*, and two universal haploid chloroplast (cpSSR), i.e. maternally inherited, microsatellite markers as described previously^39^. The final data set consisted of 730 samples for nSSR and 746 for cpSSR. Genotypes of nSSR markers were tested for null alleles using the program FreeNA^78^ finding null allele frequencies > 0.2 in 4.8% of locus x population combinations, preferentially in Southern African populations. We did not correct for null alleles, as the number and identity of null alleles is unknown. However, the results obtained with null allele corrected data are qualitatively the same (Supplementary Fig. 4). For the nSSR data set, intrapopulation genetic diversity was estimated as the total number of alleles per locus (Na), the average number of alleles per locus over loci (A_avr_), the unbiased estimate of expected (*H*_e_) and observed (*H*_o_) heterozygosity using Arlequin v.3.5.1.3^79^. The number of private alleles (A_priv_) was estimated using GDA v.1.0^80^. FSTAT v.1.2^81^ was used to calculate the inbreeding coefficient (*F*_is_) and rarified allelic richness (A_r_), thus correcting for different sample sizes by rarefaction^82^. The package genepop v.1.2^83^ was used to perform exact tests of Hardy–Weinberg equilibrium (HWE).

Population genetic structure was evaluated using a Bayesian clustering approach implemented in STRUCTURE v.2.3.4^84^. For each K ranging from one to 29 (the number of sampling sites), we performed 90 replicate runs with 100,000 steps after a burn-in period of 50,000 steps considering the model of correlated allele frequencies and admixture without prior population information^85^. The “Evanno approach”^85^ was used to identify the value of K for the uppermost hierarchical level using Structure Harvester^86^, but we also scrutinized whether results of other K allowed for a clear biologically interpretation, i.e. whether emerging clusters include several individuals who are strongly assigned to that cluster, as suggested in the STURCTURE manual^87^. Moreover, as different non-symmetric modes of model outcomes are possible in large and complex data sets^87^, we used CLUMPAK^43^ to sort out modes, align clusters across runs and calculate the consensus. We used STRUCTURE both for an analysis with only nSSR data (Fig. 1) and a combined data set of nSSR and cpSSR data (Fig. 2), thus combining all available evidence.

For the cpSSR data, we used HAPLOTYPE v.1.05^88^ to estimate the mean number of alleles per locus (N_acpSSR_), number of haplotypes detected per population (A), effective number of haplotypes (N_e_), genetic diversity (D2_sh_), haplotype richness, correcting for sample size (Rh), the number of private haplotypes (P), gene diversity within and over all populations. A parsimony network illustrating genetic relationships among haplotypes was inferred using PopArt^89^, assuming single-step mutations between alleles. For both nSSR and cpSSR, we quantified genetic differentiation with analyses of molecular variance (AMOVA) using sampling sites and clusters suggested by STRUCTURE as hierarchical levels using GenAlex v.6.^90^.

We tested for a decline of genetic variation (A_r_) with distance from the East African origin of the range expansion into West Africa, with a linear model. A hypothetical location at 0.57°N; 36.38°E between two eastern populations was chosen as the point of origin. We compared population level diversity estimates among the three phylogroups with ANOVA, followed by a TukeyHSD test.

### Geographic and environmental drivers of genetic structure

To elucidate the roles of geographic and environmental factors for genetic differentiation, we tested for isolation by distance (IBD) and isolation by environment (IBE). We generated matrices for genetic distance as pairwise *F*_ST_ (Supplementary Table 5) using GenAlex and geographic distance. Environmental distances were generated as Euclidian distances of four bioclimatic and soil variables that have been shown to best predict the current distribution of *S. senegal* in Africa (Lyam et al. 2020; Supplementary Table 6): mean temperature of the wettest quarter (Bio8), precipitation of the wettest month (Bio13)^41^, monthly variability in potential evapotranspiration (PETseasonality)^91^ and soil pH^92^. To quantify IBD and IBE, we performed Multiple Matrix Regression with Randomization (MMRR) using the R function ‘MMRR’^93^. This analysis was also done for two subranges (Sudano-Sahelian + Zambezian and Zambezian + Southern) to assess whether the same parameters are relevant in different parts of the range.

### Future projection of current climate distribution models

To assess the potential loss of intraspecific genetic diversity due to climate change, we obtained the current potential distribution for the phylogroups of *S. senegal* generated from a total of 1132 unique occurrence records and four environmental variables^27^. The occurrence records were assigned to three phylogroups at *K* = 3 (Supplementary Fig. 3). We used the result of the Bayesian Cluster analysis at *K* = 3 because the three genetic groups corresponding to the biogeographic zones of Sudano-Sahelian, Zambezian and Southern Africa were strongly genetically differentiated and spatially coherent. The current potential distribution of the three phylogroups was independently projected at a resolution of 30 s (0.93 × 0.93 km = 0.86 km^2^ at the equator) while the future projections were assessed at a resolution of 10 min (18.6 × 18.6 km = 346 km^2^ at the equator) to three IPCC climate scenarios. We use the most recently updated scenarios based on different socioeconomic assumptions, also known as the “Shared Socioeconomic Pathways” (SSPs). The SSP2 4.5 (RCP 4.5) and SSP4 6.0 (RCP 6.0) are intermediate scenarios, while the SSP5 8.5 (RCP 8.5) is the high emission scenario. All projections were estimated by ensemble SDMs implemented in Biomod2. The model accuracy was evaluated with kappa, TSS and AUC for each phylogenetic group (Supplementary Table 4). We generated continuous probabilistic maps for the current and future potential distributions of three phylogroups of *S. senegal* in Africa. To obtain final range changes, we downscaled the future projections by resampling all rasters to the resolution of the current projection and quantified absolute and relative range changes of the phylogroups.

### Research permit

The collection of plant material used in this study complied with relevant institutional, national, and international guidelines and legislation, in particular the Nagoya protocol. The following research permits were obtained for sampling for this study. Permit number: ZA/LP/88394 (issued date: 26.04.2018) for Limpopo; Permit number: MPB. 1371 (Issued date: 16.03.2018) for Mpumalanga both from South Africa. Permit number: RPIV00312018 (Issued date: 29.10.2018) for Namibia. Samples from Tanzania were received from the Herbarium, Department of Botany, University of Dar es Salam, Tanzania through collaboration (Reference number: BOT/H.3/2017; Issued date: 08.06.2017). Leaf material from six populations sampled in Ethiopia, Sudan, Mali, Mauritania, Burkina Faso and Senegal were received from Odee et al. 2012 (NERC Centre for Ecology and Hydrology, Bush Estate, Penicuik Midlothian EH26 0QB Edinburgh, UK). Populations from Nigeria, Republic of Niger and Ghana were sampled under the coverage of the Economic Community of West African States (ECOWAS) protocol, in favor of the first author.

## Supplementary Information

Supplementary Information.

## Data Availability

Genotype data of nSSR and cpSSR loci for all samples are available as supplementary file, as are the occurrence records used for SDM.
