# Supplementary material for: Climate change will disproportionally affect the most genetically diverse lineages of a widespread African tree species
Source: Sci Rep. 2022 Apr 29;12:7035. doi: 10.1038/s41598-022-11182-z (PMC9054768; doi:10.1038/s41598-022-11182-z)
Supplement: Supplementary file 1 — Supplementary Information. [file 41598_2022_11182_MOESM1_ESM.docx]

**Climate change will disproportionally affect the most genetically diverse lineages of a widespread African tree species**

P.T. Lyam^*^, J. Duque-Lazo, F. Hauenschild, J. Schnitzler, A.N. Muellner-Riehl, M. Greve, H. Ndangalasi, A. Myburgh, W. Durka

*Corresponding author: [pauliam003@gmail.com](mailto:pauliam003@gmail.com)

**Supplementary Tables** (pp 2-11)

**Supplementary Figures** (pp 12-15)

**Supplementary Table 1.** Sampling data for *Senegalia senegal* plant material analyzed in the current study. N, number of samples per population.

| Population | Lat | Long | N | Locality | Country | Biogeographic region |
| --- | --- | --- | --- | --- | --- | --- |
| DIA | 16.0000 | -15.9 | 10 | Diamenar | Senegal | Sudano-Sahelian |
| KIR | 15.3833 | -10.25 | 10 | Kirane | Mali | Sudano-Sahelian |
| MAU | 15.7333 | -8.6667 | 6 | Djigueni | Mauritania | Sudano-Sahelian |
| DIS | 13.1667 | -3.4167 | 14 | Di Sourou | Burkina Faso | Sudano-Sahelian |
| BKG | 11.0154 | -0.1994 | 13 | Bawku | Ghana | Sudano-Sahelian |
| SOK | 12.5789 | 4.97439 | 22 | Sokoto | Nigeria | Sudano-Sahelian |
| ZUR | 11.4071 | 5.23942 | 20 | Zuru | Nigeria | Sudano-Sahelian |
| RUM | 12.8741 | 7.2369 | 26 | Rumah | Nigeria | Sudano-Sahelian |
| AGU | 13.5174 | 7.66298 | 48 | Aguie | Niger | Sudano-Sahelian |
| MAR | 13.4614 | 7.10244 | 50 | Maradi | Niger | Sudano-Sahelian |
| HAD | 12.4878 | 10.0422 | 44 | Hadejia | Nigeria | Sudano-Sahelian |
| GUR | 12.6428 | 10.4534 | 21 | Guri | Nigeria | Sudano-Sahelian |
| GAS | 13.0170 | 11.0017 | 48 | Gashua | Nigeria | Sudano-Sahelian |
| GOU | 13.7063 | 11.1968 | 25 | Goudoumaria | Niger | Sudano-Sahelian |
| MDG | 11.8020 | 13.2118 | 24 | Maiduguri | Nigeria | Sudano-Sahelian |
| KOR | 12.7333 | 29.5833 | 6 | Kordofan | Sudan | Sudano-Sahelian |
| ETH | 5.98333 | 37.5333 | 10 | Arba-Minch | Ethiopia | Sudano-Sahelian |
| KIG | -6.0879 | 35.4931 | 20 | Kigwe | Tanzania | Zambezian |
| CHA | -4.8396 | 35.8314 | 20 | Chakwa | Tanzania | Zambezian |
| MBA | -6.6541 | 38.5483 | 21 | Mbala | Tanzania | Zambezian |
| MEL | -6.9367 | 37.3854 | 20 | Melela | Tanzania | Zambezian |
| VIV | -23.06779 | 29.270774 | 25 | Vivo | South Africa | Southern African/Kalahari |
| MAG | -22.724418 | 28.774937 | 25 | Magalakwena | South Africa | Southern African/Kalahari |
| BUR | -24.690596 | 30.338713 | 38 | Burgersfort | South Africa | Southern African/Kalahari |
| REH | -23.32125 | 17.064528 | 30 | Rehoboth | Namibia | Southern African/Namib |
| BRA | -21.0390 | 14.8972 | 37 | Brandberg | Namibia | Southern African/Namib |
| SOL | -24.3350 | 16.2578 | 36 | Solitaire | Namibia | Southern African/Namib |
| SPI | -21.8450 | 15.1834 | 38 | Spitzkoppe | Namibia | Southern African/Namib |
| VIN | -20.4640 | 15.3865 | 39 | Vingerklip | Namibia | Southern African/Namib |

**Supplementary Table 2.**  Analysis of molecular variance for nSSR and cpDNA in *Senegalia senegal*

|  | nSSR | | | cpSSR | | |
| --- | --- | --- | --- | --- | --- | --- |
| Source of variation | **DF** | **% Mol. var** | **F-statistics** | **DF** | **% Mol. var** | **Phi-statistics** |
| Non-hierarchical |  |  |  |  |  |  |
| Among populations | 28 | 29% | *F*_ST_ = 0.287* | 24 | 73% |  |
| Among individuals within populations | 701 | 5% | *F*_IS_ = 0.071 | 536 | 27% |  Φ_PT_=0.733* |
| Within individuals | 730 | 66% | *F*_IT_ = 0.337* |  |  |  |
| Hierarchical analysis (K=2) |  |  | *F*_ST_= 0.347* |  |  |  |
| Among clusters | 1 | 17% | *F*_RT_ = 0.171* | 1 | 43% | Φ_RT_ = 0.427* |
| Among populations within clusters | 27 | 17% | *F*_SR_ = 0.212* | 27 | 36% |  Φ_PR_ = 0.629* |
| Among individuals within populations | 701 | 5% | *F*_IS_ = 0.071 | 717 | 21% | Φ_PT_ = 0.788* |
| Within individuals | 730 | 61% | *F*_IT_ = 0.390* |  |  |  |
| Hierarchical analysis (K=3) |  |  | *F*_ST_= 0.343* |  |  |  |
| Among clusters | 2 | 20% | *F*_RT_ = 0.201* | 2 | 48% | Φ_RT_ = 0.485* |
| Among populations within clusters | 26 | 14% | *F*_SR_ = 0.178* | 26 | 30% | Φ_PR_ = 0.579* |
| Among individuals within populations | 701 | 5% | *F*_IS_ = 0.071 | 717 | 22% | Φ_PT_ = 0.783* |
| Within individuals | 730 | 61% | *F*_IT_ = 0.332* |  |  |  |
| Hierarchical analysis (K=4) |  |  | *F*_ST_= 0.334* |  |  |  |
| Among clusters | 3 | 22% | *F*_RT_ = 0.218* | 3 | 54% | Φ_RT_ = 0.541* |
| Among populations within clusters | 25 | 11% | *F*_SR_ = 0.149* | 25 | 24% | Φ_PR_ = 0.512* |
| Among individuals within populations | 701 | 5% | *F*_IS_ = 0.071 | 717 | 22% | Φ_PT_= 0.776* |
| Within individuals | 730 | 62% | *F*_IT_ = 0.381* |  |  |  |
| Hierarchical analysis (K=6) |  |  | *F*_ST_= 0.312* |  |  |  |
| Among clusters | 5 | 23% | F_RT_ = 0.234* | 5 | 68% | Φ_RT_ = 0.681* |
| Among populations within clusters | 23 | 8% | *F*_SR_ = 0.102* | 23 | 8% | Φ_PR_ = 0.249* |
| Among individuals within populations | 701 | 5% | *F*_IS_ = 0.071* | 717 | 24% | Φ_PT_ = 0.761* |
| Within individuals | 730 | 64% | *F*_IT_ = 0.361* |  |  |  |
| Hierarchical analysis (K=7) |  |  | *F*_ST_= 0.312* |  |  |  |
| Among clusters | 6 | 25% | *F*_RT_ = 0.247* | 6 | 90% | Φ_RT_ = 0.901* |
| Among populations within clusters | 22 | 7% | *F*_SR_ = 0.087* | 22 | 2% | Φ_PR_ = 0.244* |
| Among individuals within populations | 701 | 5% | *F*_IS_ = 0.071* | 717 | 7% | Φ_PT_ = 0.926* |
| Within individuals | 730 | 64% | *F*_IT_ = 0.361* |  |  |  |

Degrees of freedom (DF), Percentage of molecular variance (% Mol. Var.), overall differentiation (*F*_ST_), differentiation among clusters (*F*_RT_), differentiation among populations within clusters (*F*_SR_), differentiation among individuals within populations (*F*_IS_), differentiation within individuals (*F*_IT_) is given. A star illustrates support (*) if p ≤ 0.001. Φ_RT_, proportion of the total genetic variance that is due to the variance between clusters; Φ_PR_, proportion of the total genetic variance that is due to the variance among populations within a cluster; Φ_PT_, proportion of the total genetic variance that is due to the variance among individuals within a variant

**Supplementary Table 3**. Genetic characteristics of 29 populations of *Senegalia senegal* at nuclear and chloroplast microsatellite markers

| nSSR |  |  |  |  |  |  |  | cpSSR |  |  |  |  |  |  |
| --- | --- | --- | --- | --- | --- | --- | --- | --- | --- | --- | --- | --- | --- | --- |
| Pop | N | A_r_ | N_a_ | A_priv_ | *H*_o_ | *H*_e_ | *F*_is_ | N | A | P | Ne | Rh | Dv | D^2^_sh_ |
| DIA | 10 | 2.6 | 2.9 | 0 | 0.411 | 0.419 | 0.02 | 10 | 1 | 0 | 1 | 0 | 0 | 0 |
| KIR | 10 | 2.2 | 2.7 | 2 | 0.778 | 0.518 | -0.54*** | 10 | 1 | 0 | 1 | 0 | 0 | 0 |
| MAU | 6 | 2.6 | 2.6 | 0 | 0.722 | 0.567 | -0.31** | 6 | 1 | 0 | 1 | 0 | 0 | 0 |
| DIS | 14 | 3 | 3.7 | 3 | 0.550 | 0.548 | -0.01 | 14 | 1 | 0 | 1 | 0 | 0 | 0 |
| BKG | 13 | 3.2 | 3.7 | 1 | 0.676 | 0.565 | -0.21** | 13 | 1 | 0 | 1 | 0 | 0 | 0 |
| SOK | 22 | 3.5 | 4.2 | 2 | 0.671 | 0.596 | -0.13** | 22 | 1 | 0 | 1 | 0 | 0 | 0 |
| ZUR | 20 | 2.7 | 2.9 | 1 | 0.726 | 0.567 | -0.29*** | 20 | 1 | 0 | 1 | 0 | 0 | 0 |
| RUM | 26 | 3.3 | 4.6 | 1 | 0.535 | 0.542 | 0.02 | 26 | 1 | 0 | 1 | 0 | 0 | 0 |
| AGU | 48 | 3.4 | 5 | 0 | 0.638 | 0.627 | -0.02 | 48 | 1 | 0 | 1 | 0 | 0 | 0 |
| MAR | 50 | 3.7 | 6.5 | 3 | 0.604 | 0.578 | -0.04 | 50 | 1 | 0 | 1 | 0 | 0 | 0 |
| HAD | 44 | 3.2 | 5.1 | 1 | 0.546 | 0.554 | 0.02** | 44 | 2 | 1 | 1.05 | 0.14 | 0.05 | 0.09 |
| GUR | 21 | 2.8 | 3.6 | 1 | 0.549 | 0.486 | -0.14* | 21 | 2 | 0 | 1.21 | 0.5 | 0.18 | 0.36 |
| GAS | 48 | 3.5 | 5.6 | 2 | 0.588 | 0.543 | -0.08 | 48 | 5 | 2 | 2.07 | 1.43 | 0.53 | 6.02 |
| GOU | 25 | 3.7 | 5.1 | 0 | 0.639 | 0.601 | -0.07 | 25 | 1 | 0 | 1 | 0 | 0 | 0 |
| MDG | 24 | 3.5 | 4.4 | 1 | 0.609 | 0.599 | -0.02 | 24 | 1 | 0 | 1 | 0 | 0 | 0 |
| KOR | 6 | 2.3 | 2.3 | 2 | 0.583 | 0.475 | -0.26 | 6 | 1 | 0 | 1 | 0 | 0 | 0 |
| ETH | 10 | 4.1 | 4.8 | 1 | 0.633 | 0.662 | 0.05 | 10 | 1 | 0 | 1 | 0 | 0 | 0 |
| KIG | 20 | 3.4 | 5.2 | 14 | 0.703 | 0.692 | 0.08** | 20 | 4 | 1 | 1.71 | 1.34 | 0.44 | 1.21 |
| CHA | 20 | 4.3 | 6.6 | 17 | 0.671 | 0.691 | -0.017 | 20 | 2 | 0 | 1.34 | 0.68 | 0.27 | 0.13 |
| MBA | 21 | 4.2 | 5.6 | 8 | 0.665 | 0.609 | 0.03*** | 21 | 2 | 0 | 1.1 | 0.29 | 0.1 | 0.05 |
| MEL | 20 | 3.9 | 6.3 | 1 | 0.45 | 0.489 | -0.09 | 20 | 3 | 1 | 2.15 | 1.27 | 0.56 | 1.15 |
| VIV | 25 | 3.7 | 6.4 | 1 | 0.544 | 0.594 | 0.086*** | 25 | 3 | 0 | 1.28 | 0.67 | 0.23 | 0.12 |
| MAG | 25 | 2.3 | 3.8 | 3 | 0.395 | 0.362 | -0.095 | 25 | 1 | 0 | 1 | 0 | 0 | 0 |
| BUR | 38 | 3.4 | 6.5 | 2 | 0.379 | 0.466 | 0.19*** | 38 | 3 | 0 | 1.31 | 0.71 | 0.24 | 0.33 |
| REH | 30 | 3.4 | 5.9 | 3 | 0.4 | 0.511 | 0.22*** | 30 | 2 | 0 | 1.99 | 0.98 | 0.52 | 0.26 |
| BRA | 37 | 3.9 | 6.9 | 4 | 0.462 | 0.602 | 0.26 *** | 37 | 5 | 1 | 2.36 | 1.89 | 0.59 | 0.75 |
| SOL | 36 | 3.5 | 6.2 | 2 | 0.419 | 0.558 | 0.25*** | 36 | 5 | 0 | 3.48 | 2.23 | 0.73 | 0.87 |
| SPI | 38 | 4 | 7.5 | 4 | 0.411 | 0.587 | 0.3*** | 38 | 8 | 2 | 5.23 | 3.03 | 0.83 | 1.3 |
| VIN | 39 | 4.1 | 7.8 | 3 | 0.371 | 0.616 | 0.4*** | 39 | 6 | 1 | 4.95 | 2.9 | 0.82 | 1.29 |
| Mean | 24.9 |  |  | 2.86 | 0.5630 | 0.560 | -0.00087 | 25.72 | 2.31 | 0.31 | 1.59 | 0.62 | 0.21 | 0.48 |
| Mean: subranges | |  |  |  |  |  |  |  |  |  |  |  |  |  |
| Sudano-sahelian | | 3.14^b^ | 4.1^b^ | 1.24^b^ | 0.62^a^ | 0.56^a^ | -0.12^b^ |  | 1.35^b^ | 0.18^a^ | 1.08^b^ | 0.12^b^ | 0.05^b^ | 0.38^a^ |
| Zambezian | | 3.95^a^ | 5.93^a^ | 10^a^ | 0.62^a^ | 0.62^a^ | 0.00075^ab^ |  | 2.75^ab^ | 0.5^a^ | 1.58^ab^ | 0.90^ab^ | 0.34^a^ | 0.64^a^ |
| Southern | | 3.54^ab^ | 6.38^a^ | 2.75^b^ | 0.42^b^ | 0.54^a^ | 0.20a |  | 4.13^a^ | 0.5^a^ | 2.7^a^ | 1.55^a^ | 0.50^a^ | 0.62^a^ |

N, number of samples per location; A_r,_ rarified allelic richness (Mousadik and Petit, 1996); Na, mean number of alleles per locus per population; *H_o_* , observed heterozygosity; *H_e_* , expected heterozygosity; A_priv_, number of private alleles; *F*_is_, inbreeding coefficient (* p<0.05, ** p<0.01, *** p<0.0001); A, number of haplotypes; P, number of private haplotypes; Ne, effective number of haplotypes; Rh, haplotypic richness; Dv, genetic diversity; D^2^_sh,_ mean genetic distance between individuals. For the mean across subranges given in the lower part of the table, different letters indicate significant differences according to ANOVA and *posthoc* test. See Supplementary Table 1 for biogeographic affiliation of populations.

**Supplementary Table 4.** Accuracy values obtained from the ensemble models of three phylogeographic groups of *Senegalia senegal* habitat suitability prediction in Africa: Cohen ́s Kappa (Kappa), True Skills Statistics (TSS), Area Under the Curve (AUC), Sensitivity (true positive rate), Specificity (true negative rate).

| Ensemble model | Kappa | | | TSS | | | AUC | | | Sensitivity | | | Specificity | | | Threshold | | |
| --- | --- | --- | --- | --- | --- | --- | --- | --- | --- | --- | --- | --- | --- | --- | --- | --- | --- | --- |
|  | SUD | ZAM | SOU | SUD | ZAM | SOU | SUD | ZAM | SOU | SUD | ZAM | SOU | SUD | ZAM | SOU | SUD | ZAM | SOU |
| Mean | 0.72 | 0.69 | 0.78 | 0.85 | 0.87 | 0.88 | 0.977 | 0.974 | 0.985 | 0.965 | 0.992 | 0.951 | 0.881 | 0.876 | 0.99 | 0.576 | 0.514 | 0.602 |
| Lower Confident interval | 0.74 | 0.7 | 0.79 | 0.85 | 0.86 | 0.88 | 0.978 | 0.975 | 0.986 | 0.944 | 0.983 | 0.965 | 0.902 | 0.881 | 0.916 | 0.594 | 0.5 | 0.5 |
| Upper Confident interval | 0.7 | 0.68 | 0.77 | 0.85 | 0.867 | 0.88 | 0.975 | 0.972 | 0.984 | 0.965 | 0.992 | 0.951 | 0.88 | 0.877 | 0.928 | 0.624 | 0.549 | 0.636 |
| Median | 0.7 | 0.67 | 0.77 | 0.85 | 0.88 | 0.88 | 0.975 | 0.97 | 0.983 | 0.97 | 0.992 | 0.965 | 0.885 | 0.889 | 0.9167 | 0.624 | 0.571 | 0.537 |
| Committee averaging | 0.82 | 0.8 | 0.86 | 0.86 | 0.88 | 0.88 | 0.982 | 0.982 | 0.989 | 0.941 | 0.983 | 0.951 | 0.921 | 0.895 | 0.931 | 0.662 | 0.649 | 0.636 |
| Probability mean weight decay | 0.72 | 0.69 | 0.78 | 0.85 | 0.87 | 0.88 | 0.977 | 0.974 | 0.985 | 0.951 | 0.983 | 0.951 | 0.929 | 0.884 | 0.929 | 0.6 | 0.538 | 0.6 |

**Supplementary Table 5.** Matrix of pairwise *F*_ST_ values based on allele data at ten nuclear loci among 29 populations of *Senegalia senegal*. Most of the values estimated for both marker types were significant (*P* ≤ 0.001). See supplementary Table 4. for abbreviations of population names.

|  | DIA | KIR | MAU | DIS | BKG | SOK | ZUR | RUM | AGU | MAR | HAD | GUR | GAS | GOU | MDG | KOR | ETH | KIG | CHA | MBA | MEL | VIV | MAG | BUR | REH | BRA | SOL | SPI | VIN |
| --- | --- | --- | --- | --- | --- | --- | --- | --- | --- | --- | --- | --- | --- | --- | --- | --- | --- | --- | --- | --- | --- | --- | --- | --- | --- | --- | --- | --- | --- |
| DIA | **0.00** |  |  |  |  |  |  |  |  |  |  |  |  |  |  |  |  |  |  |  |  |  |  |  |  |  |  |  |  |
| KIR | 0.12 | **0.00** |  |  |  |  |  |  |  |  |  |  |  |  |  |  |  |  |  |  |  |  |  |  |  |  |  |  |  |
| MAU | 0.19 | 0.10 | **0.00** |  |  |  |  |  |  |  |  |  |  |  |  |  |  |  |  |  |  |  |  |  |  |  |  |  |  |
| DIS | 0.12 | 0.10 | 0.06 | **0.00** |  |  |  |  |  |  |  |  |  |  |  |  |  |  |  |  |  |  |  |  |  |  |  |  |  |
| BKG | 0.35 | 0.31 | 0.30 | 0.30 | **0.00** |  |  |  |  |  |  |  |  |  |  |  |  |  |  |  |  |  |  |  |  |  |  |  |  |
| SOK | 0.33 | 0.29 | 0.28 | 0.28 | 0.04 | **0.00** |  |  |  |  |  |  |  |  |  |  |  |  |  |  |  |  |  |  |  |  |  |  |  |
| ZUR | 0.34 | 0.31 | 0.31 | 0.28 | 0.05 | 0.05 | **0.00** |  |  |  |  |  |  |  |  |  |  |  |  |  |  |  |  |  |  |  |  |  |  |
| RUM | 0.18 | 0.11 | 0.17 | 0.10 | 0.27 | 0.25 | 0.25 | **0.00** |  |  |  |  |  |  |  |  |  |  |  |  |  |  |  |  |  |  |  |  |  |
| AGU | 0.17 | 0.11 | 0.09 | 0.05 | 0.25 | 0.24 | 0.22 | 0.07 | **0.00** |  |  |  |  |  |  |  |  |  |  |  |  |  |  |  |  |  |  |  |  |
| MAR | 0.16 | 0.09 | 0.08 | 0.05 | 0.23 | 0.22 | 0.21 | 0.06 | 0.00 | **0.00** |  |  |  |  |  |  |  |  |  |  |  |  |  |  |  |  |  |  |  |
| HAD | 0.17 | 0.11 | 0.15 | 0.10 | 0.29 | 0.27 | 0.25 | 0.01 | 0.05 | 0.04 | **0.00** |  |  |  |  |  |  |  |  |  |  |  |  |  |  |  |  |  |  |
| GUR | 0.25 | 0.19 | 0.22 | 0.18 | 0.31 | 0.28 | 0.30 | 0.06 | 0.12 | 0.11 | 0.06 | **0.00** |  |  |  |  |  |  |  |  |  |  |  |  |  |  |  |  |  |
| GAS | 0.19 | 0.13 | 0.17 | 0.13 | 0.25 | 0.23 | 0.22 | 0.03 | 0.07 | 0.06 | 0.03 | 0.04 | **0.00** |  |  |  |  |  |  |  |  |  |  |  |  |  |  |  |  |
| GOU | 0.20 | 0.13 | 0.12 | 0.11 | 0.25 | 0.24 | 0.23 | 0.08 | 0.03 | 0.03 | 0.08 | 0.12 | 0.06 | **0.00** |  |  |  |  |  |  |  |  |  |  |  |  |  |  |  |
| MDG | 0.28 | 0.24 | 0.23 | 0.21 | 0.05 | 0.02 | 0.04 | 0.19 | 0.17 | 0.15 | 0.20 | 0.22 | 0.17 | 0.18 | **0.00** |  |  |  |  |  |  |  |  |  |  |  |  |  |  |
| KOR | 0.38 | 0.31 | 0.30 | 0.23 | 0.35 | 0.33 | 0.36 | 0.17 | 0.18 | 0.18 | 0.22 | 0.29 | 0.19 | 0.14 | 0.27 | **0.00** |  |  |  |  |  |  |  |  |  |  |  |  |  |
| ETH | 0.25 | 0.18 | 0.18 | 0.20 | 0.26 | 0.25 | 0.29 | 0.15 | 0.17 | 0.16 | 0.19 | 0.17 | 0.17 | 0.14 | 0.21 | 0.23 | **0.00** |  |  |  |  |  |  |  |  |  |  |  |  |
| KIG | 0.48 | 0.46 | 0.44 | 0.44 | 0.41 | 0.39 | 0.44 | 0.41 | 0.38 | 0.37 | 0.43 | 0.46 | 0.40 | 0.36 | 0.38 | 0.47 | 0.40 | **0.00** |  |  |  |  |  |  |  |  |  |  |  |
| CHA | 0.30 | 0.27 | 0.24 | 0.27 | 0.29 | 0.29 | 0.33 | 0.27 | 0.25 | 0.24 | 0.30 | 0.30 | 0.28 | 0.23 | 0.28 | 0.32 | 0.21 | 0.36 | **0.00** |  |  |  |  |  |  |  |  |  |  |
| MBA | 0.29 | 0.24 | 0.22 | 0.27 | 0.26 | 0.27 | 0.31 | 0.26 | 0.24 | 0.23 | 0.29 | 0.32 | 0.27 | 0.23 | 0.26 | 0.30 | 0.21 | 0.38 | 0.19 | **0.00** |  |  |  |  |  |  |  |  |  |
| MEL | 0.37 | 0.33 | 0.29 | 0.31 | 0.29 | 0.31 | 0.34 | 0.29 | 0.26 | 0.25 | 0.32 | 0.36 | 0.30 | 0.25 | 0.28 | 0.31 | 0.26 | 0.39 | 0.24 | 0.10 | **0.00** |  |  |  |  |  |  |  |  |
| VIV | 0.42 | 0.38 | 0.35 | 0.38 | 0.32 | 0.31 | 0.35 | 0.36 | 0.33 | 0.33 | 0.37 | 0.38 | 0.34 | 0.33 | 0.30 | 0.42 | 0.33 | 0.39 | 0.28 | 0.32 | 0.36 | **0.00** |  |  |  |  |  |  |  |
| MAG | 0.55 | 0.52 | 0.50 | 0.50 | 0.45 | 0.43 | 0.48 | 0.48 | 0.44 | 0.43 | 0.47 | 0.49 | 0.43 | 0.45 | 0.42 | 0.57 | 0.48 | 0.52 | 0.37 | 0.44 | 0.47 | 0.18 | **0.00** |  |  |  |  |  |  |
| BUR | 0.45 | 0.42 | 0.39 | 0.40 | 0.36 | 0.35 | 0.39 | 0.39 | 0.36 | 0.36 | 0.40 | 0.42 | 0.37 | 0.37 | 0.34 | 0.46 | 0.37 | 0.44 | 0.32 | 0.37 | 0.40 | 0.045 | 0.23 | **0.00** |  |  |  |  |  |
| REH | 0.47 | 0.44 | 0.41 | 0.42 | 0.36 | 0.37 | 0.41 | 0.40 | 0.37 | 0.35 | 0.41 | 0.44 | 0.39 | 0.35 | 0.35 | 0.48 | 0.35 | 0.39 | 0.32 | 0.37 | 0.37 | 0.28 | 0.43 | 0.29 | **0.00** |  |  |  |  |
| BRA | 0.40 | 0.37 | 0.34 | 0.36 | 0.29 | 0.30 | 0.34 | 0.34 | 0.32 | 0.32 | 0.36 | 0.36 | 0.34 | 0.30 | 0.28 | 0.39 | 0.28 | 0.34 | 0.28 | 0.31 | 0.31 | 0.21 | 0.35 | 0.26 | 0.12 | **0.00** |  |  |  |
| SOL | 0.44 | 0.41 | 0.38 | 0.39 | 0.33 | 0.33 | 0.38 | 0.37 | 0.35 | 0.35 | 0.39 | 0.39 | 0.36 | 0.34 | 0.33 | 0.42 | 0.32 | 0.36 | 0.32 | 0.34 | 0.34 | 0.24 | 0.39 | 0.29 | 0.13 | 0.01 | **0.00** |  |  |
| SPI | 0.42 | 0.39 | 0.35 | 0.37 | 0.31 | 0.32 | 0.36 | 0.36 | 0.33 | 0.33 | 0.38 | 0.38 | 0.35 | 0.32 | 0.30 | 0.40 | 0.30 | 0.35 | 0.30 | 0.32 | 0.32 | 0.24 | 0.38 | 0.29 | 0.12 | 0.01 | 0.01 | **0.00** |  |
| VIN | 0.37 | 0.34 | 0.31 | 0.33 | 0.28 | 0.29 | 0.33 | 0.31 | 0.30 | 0.29 | 0.33 | 0.33 | 0.31 | 0.27 | 0.28 | 0.36 | 0.27 | 0.31 | 0.25 | 0.30 | 0.29 | 0.20 | 0.32 | 0.22 | 0.07 | 0.03 | 0.05 | 0.03 | **0.00** |

**Supplementary** Table 6. Variable importance ranking of the environmental variables used for modeling the distribution of *Senegalia senegal* in Africa. The final set of variables that were selected to run the model are highlighted in bold. The four variables were selected according to the procedure outlined in Lyam *et al*. 2020. Variable importance measures how much influence a particular variable has on the model. The higher the value, the more influence it has on the model. Probability of selection: probability that a particular variable is selected during the variable selection procedure of the modeling.

| Rank | Variable codes | Importance | Probability of selection | Variables |
| --- | --- | --- | --- | --- |
| **1** | **bio_13** | **59.65** | **1.00** | **Precipitation of wettest month^(1)^** |
| **2** | **PETseasonality** | **58.67** | **1.00** | **Monthly variability in potential evapotranspiration^(3)^** |
| **3** | **pHikcl_sl1** | **50.42** | **1.00** | **Soil pH^(2)^** |
| **4** | **bio_8** | **41.8** | **1.00** | **Mean Temperature of Wettest Quarter^(1)^** |
| 5 | bio_18 | 41.75 | 1.00 | Precipitation of Warmest Quarter^(1)^ |
| 6 | bio_3 | 39.06 | 1.00 | Isothermality (BIO2/BIO7) (* 100)^(1)^ |
| 7 | bio_9 | 32.36 | 1.00 | Mean Temperature of Driest Quarter^(1)^ |
| 8 | Ncontent | 30.03 | 0.99 | Nitrogen content^(2)^ |
| 9 | bio_15 | 28.76 | 1.00 | Precipitation Seasonality (Coefficient of Variation)^(1)^ |
| 10 | bdticm | 27.44 | 0.99 | Absolute depth to bedrock^(2)^ |
| 11 | cecsol_sl7 | 25.98 | 0.95 | Cation exchange capacity of soil at depth 2.00 m^(2)^ |
| 12 | bio_19 | 24.77 | 0.98 | Precipitation of Coldest Quarter^(1)^ |

Note: ^(1)^ Hijmans et al. 2015; ^(2)^ Hengl et al. 2017; ^(3)^ Title and Bemmels 2018.

**Supplementary Table 7.** Vouchered specimen information for populations of *Senegalia senegal* sampled for this study.

| Population code | Voucher ID | Location of voucher | Collector(s) | Date | Identified by |
| --- | --- | --- | --- | --- | --- |
| DIA | # | - | Odee et al. 2012 | - | Odee, D. |
| KIR | # | - | Odee et al. 2012 | - | Odee, D. |
| MAU | # | - | Odee et al. 2012 | - | Odee, D. |
| DIS | # | - | Odee et al. 2012 | - | Odee, D. |
| BKG | LZ 225555 | LZ, Germany | Lyam, P.T., Akpalu, S. | 20.07.2013 | Lyam, P.T |
| SOK | LZ 225553 | LZ, Germany | Lyam, P.T., Alamu, O., Olubuiyi, O. | 15.12.2012 | Lyam, P.T |
| ZUR | LZ 225554 | LZ, Germany | Lyam, P.T., Alamu, O., Olubuiyi, O. | 14.12.2012 | Lyam, P.T |
| RUM | LZ 225558 | LZ, Germany | Lyam, P.T., Kafil, I. | 22.06.2013 | Lyam, P.T |
| AGU | LZ 225552 | LZ, Germany | Moussa, M., Lyam, P.T. | 04.07.2016 | Lyam, P.T |
| MAR | LZ 225551 | LZ, Germany | Moussa, M., Lyam, P.T. | 05.07.2016 | Lyam, P.T |
| HAD | LZ 225557 | LZ, Germany | Lyam, P.T., Gado, B. | 15.06.2016 | Lyam, P.T |
| GUR | LZ 225560 | LZ, Germany | Lyam, P.T., Gado, B. | 15.06.2016 | Lyam, P.T |
| GAS | LZ 225556 | LZ, Germany | Lyam, P.T., Gado, B. | 16.06.2016 | Lyam, P.T |
| GOU | LZ 225550 | LZ, Germany | Lyam, P.T., Gado, B. | 12.07.2016 | Lyam, P.T |
| MDG | LZ 225559 | LZ, Germany | Lyam, P.T., Benisheikh, A.A. | 27.06.2013 | Lyam, P.T |
| KOR | # | RGB, Edinburgh | Odee et al. 2012 | - | Odee, D. |
| ETH | # | RGB, Edinburgh | Odee et al. 2012 | - | Odee, D. |
| KIG | HOS 5620 | DSM, Tanzania | Ndangalasi, H., Suleiman, H.O. | 31.03.2017 | Ndangalasi, H., Suleiman, H.O. |
| CHA | HOS 5623 | DSM, Tanzania | Ndangalasi, H., Suleiman, H.O. | 25.04.2017 | Ndangalasi, H., Suleiman, H.O. |
| MBA | HOS 5621 | DSM, Tanzania | Ndangalasi, H., Suleiman, H.O. | 09.04.2017 | Ndangalasi, H., Suleiman, H.O. |
| MEL | HOS 5622 | DSM, Tanzania | Ndangalasi, H., Suleiman, H.O. | 15.04.2017 | Ndangalasi, H., Suleiman, H.O. |
| VIV | N. Grobbelaar 710 | PRU, South Africa | Grobbelaar, N., | 7.2.1968 | Greve, M. |
| MAG | N. Grobbelaar 710 | PRU, South Africa | Grobbelaar, N., | 7.2.1968 | Greve, M. |
| BUR | N. Grobbelaar 710 | PRU, South Africa | Grobbelaar, N., | 7.2.1968 | Greve, M. |
| REH | LN&PL 1410 | WIND, Namibia | Lyam, P.T; Nayeni, L. | 20.11.2018 | Lyam, P.T; Nayeni, L |
| BRA | LN&PL 1414 | WIND, Namibia | Lyam, P.T; Nayeni, L | 22.11.2018 | Lyam, P.T; Nayeni, L |
| SOL | LN&PL 1412 | WIND, Namibia | Lyam, P.T; Nayeni, L | 20.11.2018 | Lyam, P.T; Nayeni, L |
| SPI | LN&PL 1413 | WIND, Namibia | Lyam, P.T; Nayeni, L | 22.11.2018 | Lyam, P.T; Nayeni, L |
| VIN | LN&PL 1416 | WIND, Namibia | Lyam, P.T; Nayeni, L | 22.11.2018 | Lyam, P.T; Nayeni, L |

**Legend**

LZ: Herbarium of the Leipzig University, Leipzig, Germany; PRU: Herbarium of the University of Pretoria, South Africa; WIND: National Herbarium of Namibia, Windhoek, Namibia; DSM: Herbarium of the Department of Botany, University of Dar es Salaam, Tanzania. The voucher (N. Grobbelaar 710, number 1733) was used as a reference for the identification and issuance of ID numbers to the samples collected from the three South African populations. Greve, M., was the identifier of the samples from these three populations. Highlighted in “**#**” represent small amount of sample material, received from already published study of Odee et al. 2012.

**Supplementary Figure 1.** Plots of mean likelihood L (K) ± SD of 90 STRUCTURE analyses per K of 730 individuals together with Delta K (Evanno et al. 2005). In the upper panel the STRUCTURE analyses were performed on the data of 10 nSSR loci while in the lower panel 10 nSSR loci plus 2 cpDNA SSR loci were used. Note that while a maximum of DeltaK indicates the uppermost hierarchical level at K=2 and K=3, increasing values of LnP(K) indicate relevant structure at higher K.

**Supplementary Figure 2.** Subrange multiple matrix regression with randomization showing patterns of (a) Isolation by distance (b) Isolation by environment (c) The relationship between geographic and environmental distances (d) Total model. Top row is Sudano-Sahelian + Zambezian ranges and bottom row is Zambezian + Southern


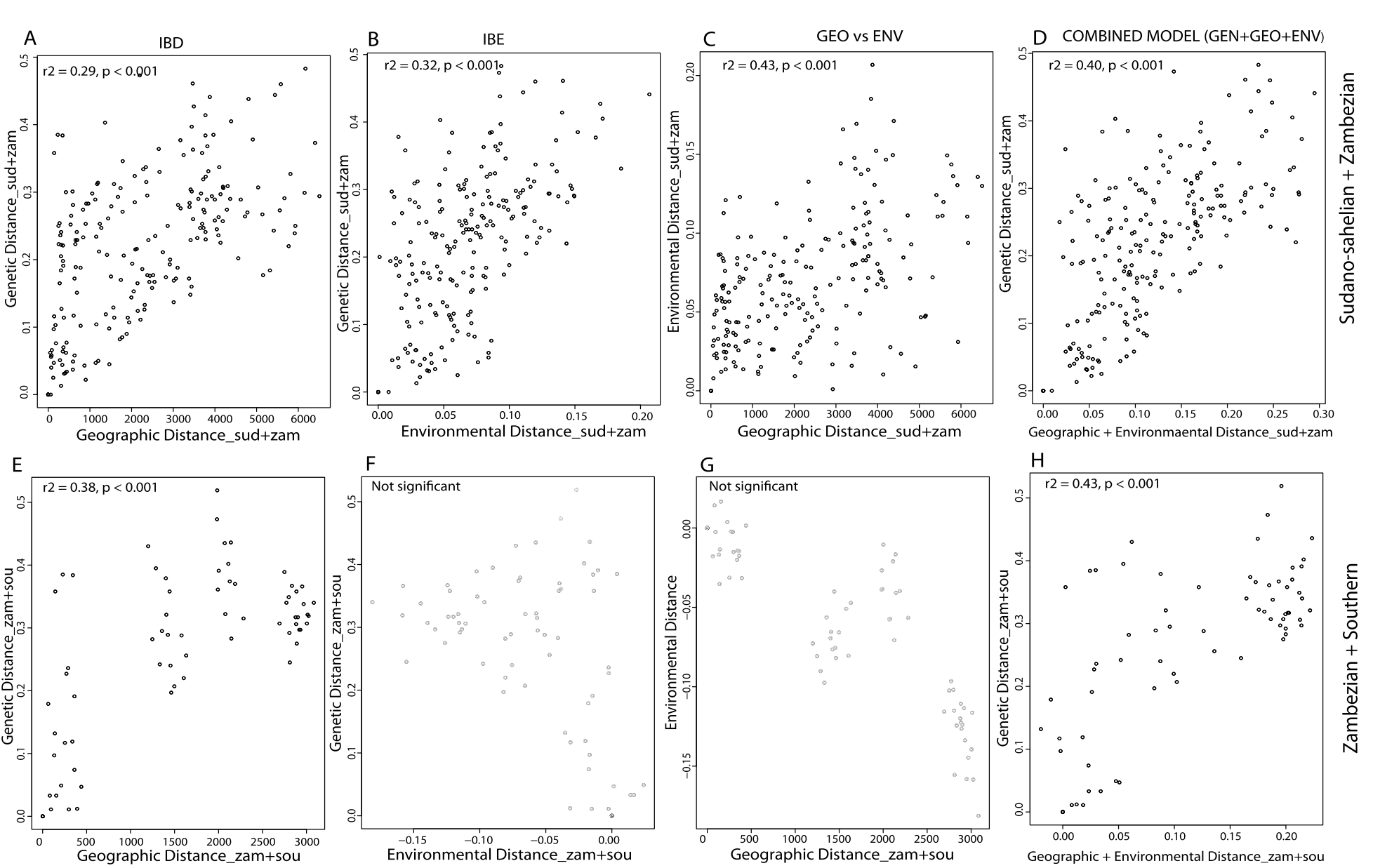


**Supplementary Figure 3.** Occurrence and potential distribution maps for three phylogeographic groups of *Senegalia senegal* in sub-Saharan Africa. (a) Occurrence records; (b) Habitat suitability for the Southern phylogroups. (c) Habitat suitability for the Sudano-Sahelian phylogroup. (d) Habitat suitability for the Zambezian phylogroup. These maps were downloaded from WORLDCLIM (Hijmans et al. 2005) and modified manually.The modeled potential distribution of *S. senegal* phylogroups were generated using ArcGIS Desktop ver. 10.5 (ESRI, 2020).


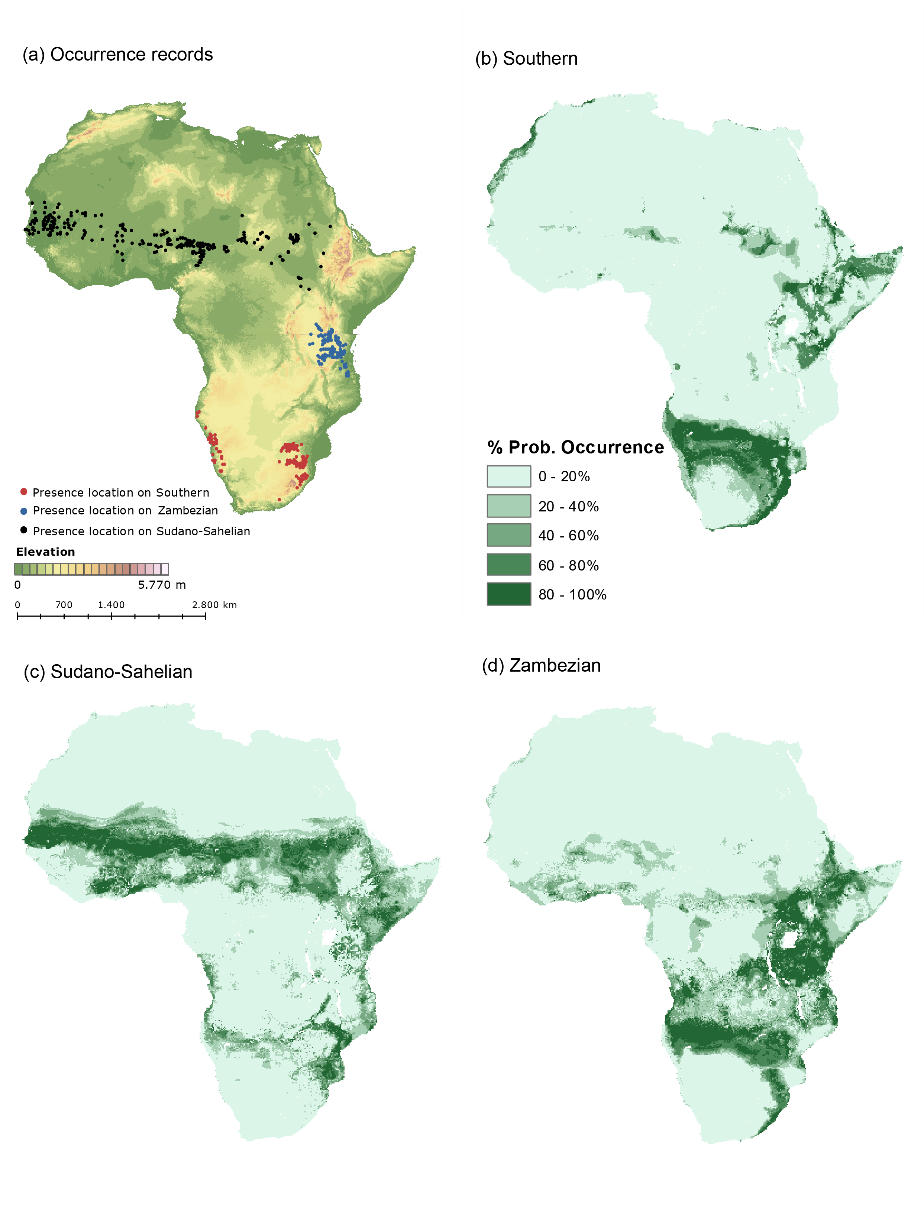


**Supplementary Figure 4**. Descriptors of genetic variation at population level calculated on the null allele-corrected nSSR data set as a function of those calculated on the original data set. Given is correlation coefficient and regression line; the red dotted line indicates the 1:1 line. If calculated on original and null allele corrected data sets, the descriptors are highly correlated between corrected and uncorrected data (except for Ho, expectedly). Consistent with the fact that null alleles are more often found in Southern Africa, which harbors higher levels of diversity, there is a trend for higher diversity gains at higher diversity levels. The main results of the paper with respect to population level diversity, i.e. a decline from East to West Africa (Fig. 3 in the ms), and higher diversity in Southern and Eastern phylogroups compared to West Africa (Tab. 1 in the ms) are not affected by null allele corrected data sets.
